# Supplementary material for: ESL-PSC Toolkit: a graphical software environment for linking shared genetic changes to convergent phenotypes
Source: Mol Biol Evol. 2026 Aug 12;43(8):msag205. doi: 10.1093/molbev/msag205 (PMC13511134; doi:10.1093/molbev/msag205)
Supplement: Supplementary file 1 [file msag205_supplementary_data.zip › Supplementary_Methods.pdf]

Supplementary Information

ESL-PSC Toolkit: a graphical software environment for linking shared genetic changes to convergent phenotypes

John B. Allard[1,2*] and Sudhir Kumar[1,2]

*Corresponding author
john.allard@temple.edu

Affiliations
1 Institute for Genomics and Evolutionary Medicine, Temple University, Philadelphia, PA 19122, USA
2 Department of Biology, Temple University, Philadelphia, PA 19122, USA

Contents:

Supplementary methods
Supplementary References

Supplementary Methods

1. Species-level SPS plots

When phenotype annotations are supplied for species in the predictions file, ESL-PSC can automatically generate species-level sequence prediction score (SPS) plots. In the current implementation, the SPS plotting workflow supports two binary summary modes shown side by side: (i) an MFS-filtered panel using only the lowest 5% of input-RMSE models (Allard et al. 2025) and (ii) a species-level aggregate panel in which SPS values are averaged across all fitted models for each species. For violin plots, SPS values are clipped to the interval [−1, 1] for display, whereas the KDE version uses the underlying values directly. Classification accuracy, true-positive rate, true-negative rate, and balanced accuracy are reported from the displayed data subset.

1.1. Photosynthesis example used in Figure 3A

Figure 3A uses the bundled C3/C4 photosynthesis demonstration, including the bundled single-combination species-groups file, protein alignments, prediction alignments, and phenotype annotations. ESL-PSC was run with partner-only deletion cancellation and a 20 × 20 log-spaced grid for both sparse-group-lasso penalty parameters from 0.01 to 0.99, with median group-penalty scaling, a maximum of 100 iterations, and a minimum of two usable pairs per alignment. The plotted points are species-level mean SPS values computed by averaging the available predictions across all completed fitted models for each of the 52 test species. The figure therefore shows the species-averaged summary panel of the SPS plotting workflow, not the lower-5%-RMSE panel. Violin distributions were drawn from those species-level mean SPS values, with the displayed values clipped to the interval [−1, 1].

2. Automatic pair selection

2.1. Overview

Automatic pair selection starts from a rooted phylogeny and a species-to-phenotype mapping. The goal is to define non-overlapping convergent-versus-control pairs suitable for ESL-PSC species-groups files. The software supports both binary and continuous phenotypes.

For binary phenotypes, species are assigned directly to the convergent (+1) or control (−1) class. For continuous phenotypes, two workflows are available. The first temporarily binarizes the phenotype values using user-specified lower and upper thresholds; species above the upper threshold are treated as convergent, species below the lower threshold are treated as control, and middle species are ignored for the purpose of pair construction. Thresholds may be set directly or derived from symmetric quantile tails. The second workflow is a local percent-contrast selector for strictly positive continuous phenotypes.

In the thresholded workflow, candidate pairs are generated by traversing terminal leaves in tree order and recording adjacent transitions between convergent and control species. Each candidate pair stores the two species, the common ancestor of the pair, and the phylogenetic distance between the species. Candidate pairs are then sorted by increasing phylogenetic distance before tie resolution and blocking are applied.

To avoid phylogenetically overlapping training sets, once a pair is accepted its ancestral clade is marked as blocked, and later candidate pairs whose ancestral nodes are descendants or ancestors of a blocked node are rejected.

2.2. Pair-selection methods

The software currently supports the following automatic pair-selection methods.

2.2.1. simple_deterministic / default

This is the original deterministic adjacent-transition behavior. Candidate pairs are generated from neighboring opposite-state leaves in tree order and sorted by phylogenetic distance. Within an ambiguous local ancestor containing multiple convergent or multiple control species, the first valid deterministic choice is taken. This option preserves historical ESL-PSC behavior and is the simplest fully deterministic mode.

2.2.2. shortest

This method uses the same candidate-pair generation and ancestor blocking as the default mode, but resolves ambiguous local ancestor clades by choosing the convergent-control duo with the shortest phylogenetic distance. This is useful when the user wants the most locally matched pairs among several valid choices.

2.2.3. longest

This method requires an alignments directory. When a candidate ancestor contains multiple convergent or control species, the selected species are the ones with the strongest sequence-length support, estimated from the longest usable aligned sequence evidence across the available alignments. The purpose is to prefer species whose alignments are likely to retain more informative sites after deletion cancellation.

2.2.4. contrast

This method is intended for thresholded continuous phenotypes. After the continuous trait has been temporarily binarized into convergent and control groups, ambiguous candidate pairs are resolved by maximizing trait contrast between the selected convergent and control species. The topological blocking rule is then applied in the same way as for the other methods.

2.2.5. composite

This method combines trait contrast, phylogenetic proximity, and sequence support. It requires an alignments directory because sequence-length evidence is part of the score.

For each ambiguous candidate ancestor, all possible convergent-control duos within that ancestor are evaluated. Let the phenotype values of the two species be v_a and v_b, with $v_a > v_b$. The phenotype contrast term is

$$T = (v_a - v_b) / S_{\text{global}}$$

where S_{global} is a robust scale estimate defined as the maximum of three quantities: 1.4826 times the median absolute deviation, the central 80% range divided by 2.563, and a small numerical floor. Sequence support is represented by the harmonic mean of the two species' sequence-length support values. A gating term G_{len} is then applied:

- if the duo-length ratio r relative to the median support is at least 0.90, $G_{\text{len}} = 1.0$
- if r is at most 0.50, $G_{\text{len}} = \varepsilon$, where $\varepsilon = 0.05$
- otherwise G_{len} is interpolated linearly between ε and 1.0

The composite score is

$$S = T\,G_{\text{len}}$$

For each candidate ancestor, all duos with composite score within a tie window of the best score are retained, where the tie window is the more permissive of an absolute difference of 0.15 or a relative difference of 5%. The final chosen duo is then selected from that tie set by shortest phylogenetic distance, then by larger raw trait contrast, then by larger sequence support.

2.2.6. random

This method uses the same candidate generation and blocking rules but resolves local ambiguities randomly. A seed may be provided for reproducibility. The CLI can also generate multiple random species-groups files in one command by incrementing the seed across runs.

2.2.7. pct_contrast

This method is available only for continuous phenotypes with strictly positive values and does not support alternates. It does not rely on temporary binarization. Instead, all ordered species pairs with unequal positive phenotype values are considered. For each pair, the species with the larger value is treated as the candidate convergent species and the lower-valued species is treated as the candidate control species. Let these values be v_{upper} and v_{lower}, respectively. The percent contrast is computed as

$$((v_{upper} - v_{lower}) / v_{lower}) \times 100$$

Candidate pairs are sorted by increasing phylogenetic distance, then by decreasing percent contrast. Pairs are accepted greedily if their percent contrast exceeds the user-defined minimum threshold and their ancestral clades do not overlap any previously selected ancestor. The GUI can preview a threshold sweep by counting how many non-overlapping pairs would be selected at each tested percent-difference cutoff.

2.3. Alternates and combination control

Except for pct_contrast, the GUI can also store alternate species choices within an accepted ancestor when multiple valid convergent or control species remain available. These alternates can be capped so that the total number of implied species combinations does not exceed a user-defined maximum.

3. Site Counter

3.1. Inputs and overall design

Site Counter scans each alignment gene-by-gene using the same species-combination structure used by ESL-PSC. Species combinations can be read directly from a species-groups file or, optionally, converted into all 2 × 2 pair combinations. The method can use either (i) a designated extant outgroup species or (ii) an ancestral state set reconstructed at the MRCA of the analysis species from a tree file.

The rationale for comparing Site Counter to the original ESL-PSC ranking on the echolocation example, and the earlier CCS comparison that motivated this addition, are discussed in the main ESL-PSC paper (Allard et al. 2025).

3.2. Site processing

For each alignment column within a given species combination, the method first extracts the convergent-group residues, control-group residues, and outgroup residue. Residues that are singletons across the convergent plus control species are masked. Missing values, gaps, and X/x residues are ignored in subsequent CCS checks.

When tree-based ancestral mode is used, the MRCA state for each column is represented as a set of possible residues obtained by Fitch parsimony (Fitch 1971). When --require_unambiguous_mrca is supplied, columns with ambiguous ancestral state sets are excluded from CCS detection. If ancestral mode is not used, the MRCA set falls back to the outgroup residue when the outgroup state is unambiguous at that column.

3.3. CCS-like rules

For a species combination to contribute to the true convergence count, at least two convergent species and at least one control species must be present in the alignment for that combination. A site is counted as convergent if:

1. the MRCA residue set is defined
2. the fraction of control residues matching the MRCA set is at least the user-defined minimum control-agreement threshold
3. at least one convergent residue not present in the MRCA set occurs at least twice among the convergent species

The default minimum control-agreement threshold is 1.0, which corresponds to the classical CCS-style requirement that all control residues agree with the ancestral or outgroup state (Xu et al. 2017).

The method also computes a control-convergence count by applying the same logic in the reverse direction: the convergent species must match the MRCA state, and at least one non-ancestral residue must occur at least twice among the control species.

3.4. Convergence-score sites

In addition to CCS-style site counts, Site Counter records the number of sites with convergence score ≥ 4 for the best-supported species combination in that gene. At each site, the method forms the residue-count difference vector between convergent and control species, sums the absolute values of that difference vector, subtracts the number of gaps in the compared species, floors the result at zero, and counts the site if the final score is at least 4.

3.5. Per-gene summary statistics

For each gene, Site Counter reports:

- avg_true: average CCS count across eligible true-direction species combinations
- avg_control: average control-convergence count across eligible reverse-direction combinations
- diff: avg_true - avg_control
- variable_sites: number of variable sites in the alignment after excluding all-singleton and one-singleton columns
- cs_sites_ge_4: maximum number of convergence-score ≥ 4 sites observed in any one species combination
- k_pairs: number of valid pairs in the species combination that achieved the largest cs_sites_ge_4
- per_combo_true: CCS count for each species combination
- per_combo_diff: true - control count for each species combination

3.6. Default ranking

When more than one species combination is present, the default Site Counter ranking is based on repeated top-fraction membership across combinations. Let top_frac denote the top proportion of genes counted as "top" for a given ranking metric, with default value 0.01.

Three per-combination rankings are computed:

1. true CCS count ranking
   num_combos_top_frac

2. difference ranking using true - control
   num_combos_top_frac_by_diff

3. ratio ranking using
   true / (control + 1)
   num_combos_top_frac_by_ratio

For each metric, a gene receives credit for a species combination if it falls within the top fraction for that combination, including ties at the boundary. The final default sort order is:

1. num_combos_top_frac
2. num_combos_top_frac_by_ratio
3. num_combos_top_frac_by_diff
4. avg_true
5. gene name

In the echolocation comparison shown in Figure 3, this default multi-combination ranking was used directly without additional reranking.

4. Echolocation enrichment comparison in Figure 3F-G

The echolocation comparison used the same 16-species-combination design for both ESL-PSC and Site Counter. For the ESL-PSC side, enrichment was performed on the published echolocation top-100 protein list from the original ESL-PSC result set so that the comparison remained aligned with the previously reported analysis. For the Site Counter side, the human sequence was designated as the extant outgroup, and enrichment was performed on the top 100 proteins from the default multi-combination Site Counter ranking described above.

For the original ESL-PSC echolocation analysis and the corresponding published top-ranked proteins, see Allard et al. (2025).

Both lists were analyzed with the background-aware enrichment analysis through Enrichr, using the same Orthomam background of 14,499 genes rather than a generic genome-wide

background. The queried libraries were GO Biological Process 2023, GO Molecular Function 2023, GO Cellular Component 2023, MGI Mammalian Phenotype Level 4 2024, and Human Phenotype Ontology. Significant terms were defined as those with adjusted q-value < 0.05 and overlap ≥ 5 genes. The hearing-related profile shown in Figure 3F was obtained by filtering those significant terms to names containing hearing- or auditory-related keywords, specifically hearing, auditory, cochlea, cochlear, deaf, otoacoustic, sound, or organ of corti, and then ranking the retained terms by adjusted P-value within each method.

The protein sets shown in Figure 3G were defined as the union of all overlapping genes from those retained significant hearing-related terms for each method. The Venn counts therefore summarize proteins that contributed to at least one significant hearing-related enrichment under the stated filter, rather than all proteins in the top-100 lists.

5. Response-flip null model

The response-flip null model preserves the same species combinations and alignments as the true ESL-PSC analysis, but reverses pair labels in a balanced way. For an even number of training pairs, all balanced choices of half the pairs are reversed, with mirror-image labelings collapsed so that equivalent complements are not duplicated. The resulting null combinations are then run through the standard ESL-PSC workflow and aggregated in the same way as the true combinations.

For four training pairs, each balanced null realization necessarily leaves two pairs in their original orientation and reverses two pairs. As a result, response-flip null models retain residual structure and should not be interpreted as completely signal-free random permutations. This is why AUROC values in the body-mass null comparisons can remain above 0.5.

6. Continuous-trait evaluation in the body-mass example

6.1. Data sources and dataset size

Adult body-mass values were derived from the AnAge table accessed on August 27, 2025 (de Magalhães et al. 2024). We retained Mammalia records with positive values, preferred Adult weight (g) when available and otherwise used Body mass (g), and used the median when AnAge contained multiple values for the same species. TOGA species identifiers were matched first exactly, then by the first two taxonomic name components so that infraspecific assembly labels could inherit species-level values, and finally by three manually documented current-name synonyms (Callithrix pygmaea to Cebuella pygmaea, Equus burchellii boehmi to Equus quagga, and Lyroderma lyra to Megaderma lyra). This procedure assigned body mass to 348 of the 442 species represented in at least one final alignment.

The sequence dataset was constructed from all 17,439 human-hg38-reference MultipleCodonAlignments distributed by the original TOGA/Zoonomia resource (Kirilenko et al. 2023;

https://genome.senckenberg.de/download/TOGA/human_hg38_reference/MultipleCodonAlignments/). We then generated a stricter species-level one-to-one amino-acid dataset in two passes. For every query assembly, its TOGA orthologsClassification.tsv.gz and loss_summ_data.tsv.gz records were used to retain only one-to-one projections in the Intact, Partially Intact, or Uncertain Loss classes. In the first pass across all genes, we counted retained one-to-one genes for every assembly. Exactly one assembly was then selected globally for each species by maximizing that count, with scaffold N50, contig N50, and assembly name used successively to break ties. This global selection prevented different assemblies of the same species from being mixed among genes. In the second pass, each gene retained the human reference plus qualifying projections from the selected assemblies. If more than one qualifying transcript from the selected assembly survived for a species-gene combination, that species was omitted from that gene rather than choosing a co-ortholog arbitrarily. This resulted in 17,439 gene alignments including 442 species.

For contrast pair selection, held-out filtering, and phylogenetic sensitivity analyses, we used the species phylogeny distributed with the TOGA2 human-hg38 v1 multiple-codon alignment resource (Malovichko et al. 2026; https://genome.senckenberg.de/download/TOGA2/MultiCodonAlignments/reference_human_hg38/v1/speciesTree.nh). The downloaded phylogeny contained 882 assembly-level tips. After pruning to the taxa represented in the alignment metadata and reconciling assembly labels to species, 719 tips remained. We rooted this tree on the edge separating monophyletic Atlantogenata from Boreoeutheria, dividing that edge equally between the two root descendants.

6.2. Training and evaluation design

Species in the upper and lower quartiles of the 348-species body-mass distribution were provisionally labeled high and low, respectively, and ESL-PSC's contrast selector was used to resolve ambiguous local choices by maximizing the body-mass difference while enforcing the non-overlapping-MRCA rule. This yielded four high-versus-low pairs: Gorilla gorilla gorilla versus Callithrix pygmaea, Mirounga angustirostris versus Mustela erminea, Panthera leo versus Helogale parvula, and Loxodonta africana versus Elephantulus edwardii. The true-design analysis used a 4 × 4 log-spaced grid for both sparse-group-lasso penalty parameters from 0.01 to 0.99 (16 models). The response-flip null used the same inputs and fitting settings with the built-in null-model option.

The evaluation set was reconstructed from all 340 taxa in the raw true-design and response-flip prediction outputs. For this revised evaluation, taxon names were reconciled to the more complete TOGA2 species phylogeny described above, and all descendants of the MRCA of each training pair were excluded. This criterion makes the evaluation taxa external to the training-pair clades.

After taxonomic reconciliation and exclusion of training-pair MRCA descendants, 248 sequence records remained. Some prediction records represented different assemblies or infraspecific

labels that mapped to the same species-level tip. Body mass was identical within each such group, and SPS values were averaged, yielding 239 unique species-level tips for evaluation.

Species-level mean SPS values were computed by taking the arithmetic mean of the available prediction rows across all fitted models for each sequence record and then averaging records that mapped to the same species-level tree tip. Binary evaluation used several phenotype thresholds (q25, q30, q40, and q50) to label test species as high or low body mass, excluding intermediate species under the chosen threshold. Balanced accuracy was computed at a fixed SPS threshold of 0, and AUROC was computed from the same species-level scores. For continuous evaluation, Spearman rank correlation was calculated between mean SPS and $\log_{10}$ adult body mass across the 239 corrected tips. Spearman ρ is reported as a descriptive measure of the observed monotonic association: $\rho = 0.685$ for the true design and $\rho = 0.140$ for the response-flip null. Conventional two-sided Spearman tests gave $P = 1.69 \times 10^{-34}$ and $P = 0.0304$, respectively, but these P values treat tips as independent observations. They are therefore reported only as nominal results and were not used for phylogenetically informed inference.

To account for residual covariance among the test species, we fitted phylogenetic generalized least-squares (PGLS) models with Pagel's λ estimated by maximum likelihood rather than fixed a priori (Pagel 1999; Freckleton et al. 2002; Revell 2010). This analysis is complementary to PSC training: pair contrasts constrain model fitting, whereas PGLS evaluates covariance among held-out residuals. Estimating λ allows the evaluation model to range between independent residuals ($\lambda = 0$) and the tree-derived correlation structure ($\lambda = 1$). For a rank-based PGLS analogue of the Spearman analysis, both $\log_{10}$ body mass and SPS were rank-transformed before fitting. The reported slope P value tests the null hypothesis that the SPS slope is zero conditional on the estimated phylogenetic covariance. Models were fitted in R 4.5.3 using ape 5.8-1 and nlme 3.1-169.

The primary PGLS used the rerooted species phylogeny and the normalized phylogenetic correlation implemented by ape::corPagel. For two tips, the off-diagonal correlation is Pagel's λ multiplied by their shared root-to-MRCA branch length and divided by the square root of the product of their root-to-tip branch lengths; diagonal elements are one. Thus, a longer terminal branch reduces a tip's residual correlation with its relatives, while normalization keeps marginal residual variance equal across tips. The true design yielded a positive rank-based association (coefficient = 0.1800, SE = 0.0473, $t = 3.809$, $P = 1.78 \times 10^{-4}$, estimated $\lambda = 0.9726$), whereas the response-flip null association was not supported (coefficient = 0.0309, SE = 0.0335, $t = 0.923$, $P = 0.357$, estimated $\lambda = 0.9862$). To assess the equal-marginal-variance assumption, we also fitted a non-normalized Brownian variance-covariance model with Pagel's λ estimated. Each diagonal element retains that tip's root-to-tip branch length, and each off-diagonal element is Pagel's λ multiplied by the shared branch length. The inferential conclusion was concordant under this alternative covariance treatment (Supplementary Table 1).

For a time-scaled sensitivity analysis, the rerooted species phylogeny was converted to an ultrametric tree with RelTime in MEGA-CC 12.0.1 and only then pruned to the 239 test species

tips (Tamura et al. 2012; Kumar et al. 2024). Pagel's λ was re-estimated for each RelTime model. Rank PGLS on the RelTime tree supported the true-design association (coefficient = 0.1946, SE = 0.0478, P = 6.42 × 10^{-5}, estimated λ = 0.9731) but not the response-flip null association (coefficient = 0.0257, SE = 0.0338, P = 0.448, estimated λ = 0.9870). Across both ranked and plotted-value analyses, the inference was concordant under normalized correlation on the non-ultrametric species phylogeny, non-normalized Brownian covariance, and relative-time branch lengths (Supplementary Table 1).

Supplementary Table 1. Sensitivity of the body-mass association to response scale and phylogenetic covariance treatment. Entries are two-sided P values for the SPS slope; Pagel's λ was estimated separately for every model.

Scale and covariance treatment	True design P	Response-flip null P
Ranked; normalized species-phylogeny Pagel λ	1.78 × 10^{-4}	0.357
Ranked; non-normalized species-phylogeny Brownian V + λ	1.90 × 10^{-5}	0.384
Ranked; RelTime Pagel λ	6.42 × 10^{-5}	0.448
Plotted values; normalized species-phylogeny Pagel λ	0.00118	0.710
Plotted values; non-normalized species-phylogeny Brownian V + λ	1.97 × 10^{-4}	0.693
Plotted values; RelTime Pagel λ	9.22 × 10^{-4}	0.962

7. Runtime benchmark

The runtime benchmark in Figure 3B compared the legacy Python-centered ESL-PSC path with the unified Rust implementation on the echolocation design. Both implementations used the same 16 species combinations, partner-only deletion cancellation, median group-penalty scaling, no prediction output, and a 20 × 20 log-spaced lambda grid from 0.01 to 0.99. Nested input sets comprised the first 32, 100, 316, and 1,000 Orthomam FASTA files in filename-sorted order. The timed region covered each complete command-line analysis, and one Python run followed by one Rust run was recorded at each input size. Both commands were run with a soft file-descriptor limit of 1,024 to avoid the historical Python preprocessor pathology that occurs under extremely high open-file limits. Recorded wall-clock times for Python versus Rust were 57.963 versus 1.771 s for 32 alignments, 191.199 versus 2.018 s for 100, 593.306 versus 5.448 s for 316, and 1,814.313 versus 17.510 s for 1,000, corresponding to speedups of 32.7×, 94.7×, 108.9×, and 103.6×, respectively.

8. Use of artificial intelligence tools

Agentic artificial intelligence tools (OpenAI Codex agents powered by frontier AI models) were used to assist with software development, software testing, data preparation, implementation and checking of statistical analyses, technical aspects of figure preparation, and technical documentation. The authors reviewed all agent-assisted code and other work; extensively tested the software manually; and verified results against the underlying data and analysis outputs. The authors made all scientific and interpretive decisions and take full responsibility for the final work.

Supplementary References

Allard JB, Sharma S, Patel R, Sanderford M, Tamura K, Vucetic S, Gerhard GS, Kumar S. 2025. Evolutionary sparse learning reveals the shared genetic basis of convergent traits. Nat Commun. 16:3217.

de Magalhães JP, Abidi Z, Dos Santos GA, Avelar RA, Barardo D, Chatsirisupachai K, Clark P, De-Souza EA, Johnson EJ, Lopes I, et al. 2024. Human Ageing Genomic Resources: updates on key databases in ageing research. *Nucleic Acids Res.* 52:D900–D908.

Fitch WM. 1971. Toward defining the course of evolution: Minimum change for a specific tree topology. Syst Biol. 20:406–416.

Freckleton RP, Harvey PH, Pagel M. 2002. Phylogenetic analysis and comparative data: A test and review of evidence. Am Nat. 160:712–726.

Kirilenko BM, Munegowda C, Osipova E, Jebb D, Sharma V, Blumer M, Morales AE, Ahmed A-W, Kontopoulos D-G, Hilgers L, et al. 2023. Integrating gene annotation with orthology inference at scale. Science. 380:eabn3107.

Kumar S, Stecher G, Suleski M, Sanderford M, Sharma S, Tamura K. 2024. MEGA12: Molecular Evolutionary Genetic Analysis Version 12 for adaptive and green computing. Mol Biol Evol. 41:msae263.

Malovichko YV, Bein B, Gonzales-Irribarren A, Leushkin E, Hilgers L, Stephen A, Yi X, Albertini M, Stadager T, Zumpt M, et al. 2026. Accurate, comprehensive gene annotation and ortholog identification across thousands of vertebrate genomes with TOGA2. *bioRxiv* 2026.06.30.735536. doi:10.64898/2026.06.30.735536.

Pagel M. 1999. Inferring the historical patterns of biological evolution. Nature. 401:877–884.

Ranwez V, Douzery EJP, Cambon C, Chantret N, Delsuc F. 2018. MACSE v2: Toolkit for the alignment of Coding Sequences accounting for frameshifts and stop codons. Mol Biol Evol. 35:2582–2584.

Revell LJ. 2010. Phylogenetic signal and linear regression on species data. Methods Ecol Evol. 1:319–329.

Tamura K, Battistuzzi FU, Billing-Ross P, Murillo O, Filipski A, Kumar S. 2012. Estimating divergence times in large molecular phylogenies. Proc Natl Acad Sci USA. 109:19333–19338.

Xu S, He Z, Guo Z, Zhang Z, Wyckoff GJ, Greenberg A, Wu C-I, Shi S. 2017. Genome-Wide Convergence during Evolution of Mangroves from Woody Plants. Mol Biol Evol. 34:1008–1015.
